# Graphites for Pyrosis

**Published:** 1895-06

**Authors:** 


### GRAPHITES FOR PYROSIS.

A young, tall lady of nineteen years suffers from pyrosis, which attacks her at any time, so that she is hardly able to go into society, and thus become melancholic. She also suffers from chronic constipation, palpitations, worse when ascending, when she also complains of difficulty of breathing. Goallon thought at first on Bismuth, which is indicated in many cases of chlorosis, but finally settled on Graphites$^{12}$, which at first aggravated and then cured. She also acknowledged that riding in a carriage had always produced pyrosis, and one finds this aggravation by riding in a carriage sometimes in chlorosis.—*Populäre Zeitsch, f. H.* 12, 91.

